# The Context Dependency of the Self-Report Version of the Strength and Difficulties Questionnaire (SDQ): A Cross-Sectional Study between Two Administration Settings

**DOI:** 10.1371/journal.pone.0120930

**Published:** 2015-04-17

**Authors:** H. Hoofs, N. W. H. Jansen, D. C. L. Mohren, M. W. J. Jansen, I. J. Kant

**Affiliations:** 1 Department of Epidemiology, CAPHRI School for Public Health and Primary Care, Faculty of Health, Medicine and Life Sciences, Maastricht University, Maastricht, The Netherlands; 2 Academic Collaborative Centre for Public Health Limburg, Public Health Service Southern Limburg, Geleen, The Netherlands; 3 Department of Health Services Research, CAPHRI School for Public Health and Primary Care, Faculty of Health, Medicine and Life Sciences, Maastricht University, Maastricht, The Netherlands; Pennsylvania State University, UNITED STATES

## Abstract

**Background:**

The Strength and Difficulties Questionnaire (SDQ) is a screening instrument for psychosocial problems in children and adolescents, which is applied in “individual” and “collective” settings. Assessment in the individual setting is confidential for clinical applications, such as preventive child healthcare, while assessment in the collective setting is anonymous and applied in (epidemiological) research. Due to administration differences between the settings it remains unclear whether results and conclusions actually can be used interchangeably. This study therefore aims to investigate whether the SDQ is invariant across settings.

**Methods:**

Two independent samples were retrieved (mean age = 14.07 years), one from an individual setting (N = 6,594) and one from a collective setting (N = 4,613). The SDQ was administered in the second year of secondary school in both settings. Samples come from the same socio-geographic population in the Netherlands.

**Results:**

Confirmatory factor analysis showed that the SDQ was measurement invariant/equivalent across settings and gender. On average, children in the individual setting scored lower on total difficulties (mean difference = 2.05) and the psychosocial problems subscales compared to those in the collective setting. This was also reflected in the cut-off points for caseness, defined by the 90th percentiles, which were lower in the individual setting. Using cut-off points from the collective in the individual setting therefore resulted in a small number of cases, 2 to 3%, while ∼10% is expected.

**Conclusion:**

The SDQ has the same connotation across the individual and collective setting. The observed structural differences regarding the mean scores, however, undermine the validity of the cross-use of absolute SDQ-scores between these settings. Applying cut-off scores from the collective setting in the individual setting could, therefore, result in invalid conclusions and potential misuse of the instrument. To correctly apply cut-off scores these should be retrieved from the applied setting.

## Introduction

Prevalence rates of childhood psychosocial and behavioural problems are high, and if untreated potentially persistent into later life [1,2]. Their consequences, both individual and societal, are among other aspects associated with an increased risk of long-term work disability and psychiatric disorders in young adults [3,4]. Early detection of children who are at risk for developing psychopathology is therefore crucial, as this enables early social medical counselling which enhances prognosis [5,6]. To detect these children, valid and reliable screening instruments are crucial.

The Strength and Difficulties Questionnaire (SDQ) is a questionnaire developed to screen for psychopathology in children and adolescents [7]. The widespread use of the SDQ as a screening instrument is attributed to several characteristics, such as its user-friendliness, briefness (25 items), inclusion of positive attribute items, and the availability for different informants (e.g. teacher, parent, youth) [8–11].

Due to these characteristics the SDQ is frequently used in child mental health research [12]. Its application can roughly be divided into two settings, the “individual” and the “collective” setting. The individual setting can be seen as the clinical application of the SDQ (e.g. preventive child healthcare). In such a setting the instrument facilitates the decision making, by a healthcare professional, regarding an individual. In this setting the SDQ is especially suitable for early detection purposes [13–15]. In contrast, the collective setting is characterised by the aggregation of information across a population. In such a setting the SDQ is used to characterize the overall well-being of populations, for health policy-making and planning, or to compare these populations across countries [16–18]. The collective setting also covers the research regarding the relation of the SDQ with other health-related outcomes or its psychometric properties [9,19–23].

Based on the research of the foregoing decade it seems evident that the SDQ is an outstanding questionnaire for use in both individual and collective settings. However, the interchangeability of scores (e.g. cut-off points) and conclusions between these settings has never been studied so far. If this is hampered it could violate the cross-use of scores and results between settings. The goal of this study is therefore to investigate whether the SDQ is invariant across administration settings.

The most prominent aspect that differs between the settings, and therefore possibly violating a valid comparison, is its level of anonymity. Naturally the assessment in an individual setting is not anonymous, as it is otherwise impossible to relate the score to the individual. The term confidential, instead of non-anonymously, is preferred to stress that results are only available for the health professional involved [24]. Nevertheless an indicator is needed for the health professional to relate the questionnaire to the individual (e.g. national service number or handing it in in-person). In the collective setting, however, assessment can be completely anonymously [25].

Anonymity is often believed to enhance the trustworthiness of answers, as the pressure for social desirable responses is diminished [26]. This social desirability bias (SDB) refers to the act of misrepresentation of an individual in order to be perceived more positively by others. This may lead to exaggeration of positive behaviour and understating of negative behaviour [27]. The effect of SDB, however, does not only increase when the condition of assessment becomes less private, but also when questions are more sensitive [25,28]. The influence of gender should also be taken into account, as the effect of anonymous assessment on SDB varies between males and females depending on the topic of the questionnaire [27]. Studies focusing on adolescents are, however, limited and show diverse results which are modest at best [29,30]. In all these studies regarding SDB the distinction between the confidential and anonymous group lies mainly in whether the questionnaires are identifiable. In individual settings, questionnaires are however not merely identifiable, they will in fact *be* identified as elevated scores can elicit possible implications such as a follow-up or referral. Assuming that these possible consequences are known (and unwanted), this could further enhance the SDB in the individual setting in order to remain “off the radar”.

Valid conclusions regarding structural differences of the SDQ between settings, due to SDB, are only possible when there is measurement equivalence/invariance (ME/I). ME/I states that an instrument measures the same construct across different populations or settings [31]. That is, individuals who have the same standing on the measured construct should receive the same score irrespective of aspects unrelated to this score, leaving aside general inaccuracy [32,33]. If ME/I does not hold, it is not possible to unambiguously interpret whether structural differences are true differences or merely artefacts of measurement bias [34]. Or as Vandenberg et al. [33] state, in such a case ”a group mean comparison may be tantamount to comparing apples and spark plugs (p. 9).” In contrast to ME/I, structural differences reflect variation on the overall construct such as differing mean scores [31]. For groups with the same standing on a construct, structural differences should be absent [35].

For the valid cross-use of the SDQ between collective and individual settings, at least ME/I have to hold. Otherwise the connotation of the SDQ would differ between settings. Extrapolation of conclusions between settings will therefore also be hampered, prohibiting the use of the SDQ in a specific setting based on findings from other settings. When ME/I does hold, but structural mean differences exist, the picture becomes more nuanced. Although in that case the SDQ has the same connotation across settings, these differences violate comparisons regarding absolute scores between collective and individual settings.

Cut-off scores, along with mean scores, might additionally be susceptible for structural differences. In the early publications of the SDQ [7,15,36], using a mixture of settings, it was shown that children falling within the highest 10% of SDQ scores had increased odds of any DSM-IV diagnoses or being a psychiatric case. Subsequently, in collective setting research, this 10% criterion was used to create cut-off scores to identify the high risk group for various nation-wide samples [16,37,38]. As it is shown that the discriminatory power of these cut-off scores is satisfactory, one of the purposes of cut-off scores is to facilitate clinical decision making in individual settings [13,39]. The cut-off scores used in individual settings are often adopted from collective settings as normative data usually stems from these settings [40]. As cut-off scores are based on the same underlying distribution as mean scores they would also suffer from structural differences between settings. Therefore the legitimacy of transferring cut-off scores between settings is questionable when structural differences are present. As this distortion can have massive implications for actual practice, since the interpretation of “a case” would be biased, the legitimacy of this procedure will receive special attention in this paper.

The main question of this article therefore is:

- Is the self-report version of the SDQ invariant whether it is administered in an individual or collective setting?

This main question can be divided into the following sub-questions:

- Does ME/I hold across settings?
- Are there structural differences across settings?
- What is the influence of gender on ME/I and structural differences between and within settings?
- How does possible invariance between settings influence the validity of the cross-use of cut-off points?

## Methods

### Data Collection

Data from an individual and a collective sample were included to analyse the impact these different settings may have on the SDQ. Data for both settings was collected in the southern region of Limburg, the Netherlands. In contrast to other regions in the Netherlands, the area is characterized by: Its variation of rural and urban areas; a shrinking and aging population; a relatively low social economic status (SES); and, a small proportion of people with a non-western ethnic background (3%) and relative high proportion with a, non-Dutch, western ethnic background (16%) [41]. Data was collected by the Public Health Service of South-Limburg. Their services include, among others, child immunisation, preventive child healthcare (PCH) programs, and providing municipalities with epidemiological data for policy purposes.

### Individual setting

For the individual setting, data from the PCH program is used. All children who live in the Netherlands are automatically included in this program. The PCH is regulated through national regulation and resources, while the financing takes place by local governments without any parental costs [42].

Information from the screening assessment during the second year of secondary school is used in the current study. This is the final of 14 screening assessments spread across different stages in the child’s development, with more frequent screening assessments early in life. During the screening (20–30 minutes) a specially trained youth health care physician assesses the psychosocial and physical health of the child, but also pays attention to the socio emotional development of the child or other issues related to the well-being of the child. If needed the youth health care physician can advise a possible follow-up or referral to secondary or tertiary care. During the screening it is optional for a parent or guardian to accompany the child. The procedure has an opt-out approach, for which the responsibility lies with the parent(s) or guardians(s) of the child and the child themselves. Only information from screenings in regular secondary schools was used, excluding special needs education schools. There were no additional inclusion or exclusion criteria.

Approximately two weeks prior to the screening a questionnaire was sent to the child. This paper-and-pencil self-administered questionnaire (SAQ), used to facilitate the screening, consisted of questions regarding medical history, psychosocial development (using the SDQ), and substance use. The questionnaire was filled in at home and handed in during the screening assessment, in which the youth health care physician reviewed the questions and computed the total difficulties score for the SDQ. Eventually all questionnaires were scanned and processed to obtain a database for each scholastic year. Screening took place at, and during, school. For logistical reasons children were invited and scheduled school-wise. The screenings took place during the whole school year (median = March).

For the current study the data was anonymized. Data from the two most recent years of data gathering were included. This resulted in the inclusion of the school years 2010/11 and 2011/12. Circa 90% of the eligible children were present during this screening [41]. The sample included 6,594 available questionnaires. The mean age of the children was 14.26 years (SD = 0.62 years), with 51.30% of the sample being female.

### Collective setting

For the collective setting information from the National Youth Monitor was used. This monitor was used to inform policymakers and researchers about the situation of the youth in the Netherlands covering several domains, such as health and welfare [43]. The SDQ was included as indicator of psychosocial well-being of the youth. For the present study questionnaires from 2009 were used.

Administration took place in regular classes in the second year of secondary school. The intended mode of assessment was a computerized self-administered questionnaire (CSAQ). Due to technical issues this was not always possible, which led to a minority of cases which were given a SAQ (17%). Both modes were included in the sample. Administration took place in the last quarter of 2009 for all children (median = November). The procedure has an opt-in approach, in which the parent(s) or guardians(s) of the child had to give informed consent. Only information from regular secondary schools was used, excluding special needs education schools. There were no additional inclusion or exclusion criteria.

The response rate for the collective setting was approximately 70%. This resulted in 4,613 available questionnaires. The mean age of the children was 13.80 years (SD = 0.57 years), with 50.01% of the sample being female.

### Ethical statement

Research in this article involves participants who were part of the usual governmental (preventive) healthcare program attending regular secondary schools. Two methods were used to collect the data. The first method involved the retrospective patient files (individual setting). Information was anonymized and de-identified by the Public Health Service of South-Limburg before it was received and reviewed by the authors. Retrospective research/research with patient files does not fall under the scope of the Human Subjects Act (Wet medisch-wetenschappelijk onderzoek met mensen; WMO) as the research subject is not physically involved in the research. The second method involved the use of anonymous questionnaire data (collective setting), with informed consent given by the caregiver of the child. This does not require WMO approval since does not utilize any invasive techniques, substance administration or psychological manipulations. Since both methods of data collection do not require any WMO approval by the Dutch law, the approval of an ethical commission is not needed and therefore the CCMO was not contacted for a waiver or approval [44]. None of the authors played any part or had any interaction with the human participants in the two methods of data collection. Both strategies were furthermore registered at the Personal Data Protection Act (Wet bescherming persoonsgegevens, Wbp) [44].

### Measures

#### SDQ

The SDQ comprises 25 items spread equally across 5 subscales; emotional symptoms, conduct problems, hyperactivity/inattention, peer problems, and pro-social behaviour [7,45,46]. Each item gives a statement regarding negative or positive behaviour (e.g. “I worry a lot”) which can be scored on a three point Likert scale (“not true”, “somewhat true”, or “certainly true”). Five items were positively worded and were therefore reverse coded. Besides a score on each subscale, ranging from 0–10, a total difficulties score is also computed, ranging from 0–40, by summing the scores from the emotional symptoms, conduct problems, hyperactivity/inattention and peer problems subscales. Higher scores indicated elevated problems on the total difficulties score and all subscales, except for the pro-social behaviour subscale where higher scores indicated higher levels of pro-social behaviour. Further information regarding the computation of the SDQ can be found on *www*.*sdqinfo*.*org*. For the current study the Dutch self-report version was used [14].

#### Demographic characteristics

Age, gender, and home situation were retrieved from the questionnaires administered in the individual and collective setting. Social economic status (SES) and domestic area were derived from postal codes which were present in the questionnaires in both settings. SES was computed using a national database which provided a SES score for each postal code area (range = -7.3 to 3.2). These scores were based on a combination of several aspects such as education, income and employment status. As these scores represent averages they should not be seen as individual indicators, as they merely serve descriptive purposes [47]. Domestic area was defined using the classification of Statistics Netherlands. Areas with more than 1,000 addresses per square kilometre, were classified as urban while areas with a lower density were classified as rural [48]. Ethnic background was also defined in accordance with the classification system of Statistics Netherlands, in which both parents had to be born in the Netherlands to be classified as Dutch. If both the mother and father were born in a foreign country, the country of birth of the mother determined the nature of the ethnic background (western vs. non-western). If only one of the two parents was born in a foreign country this parent determined the nature of the ethnic background [49].

### Statistical analyses

Cronbach’s α and greatest lower bound (glb) were calculated as reliability coefficients. Although glb seems most appropriate, Cronbach’s α was also reported to enhance comparability with previous studies. Since the glb was always larger than the Cronbach’s α of a scale, they were reported as a range [50].

Confirmatory Factor Analysis (CFA) was used to test whether the SDQ was invariant across settings. The original hypothesized 5-factor model proposed by Goodman [15] was used including correlations between all factors. As all items were rated on a 3-point Likert scale they were handled as categorical data. The weighted least square means and variance adjusted (WLSMV) estimator was selected. This estimator has proven to be preferred over maximum likelihood for a broad range of sample sizes when handling ordered, especially asymmetrical, categorical data with few categories [51–53]. To evaluate model fit the Comparative Fit Index (CFI), Tucker-Lewis Index (TLI), and Root Mean Square Error of Approximation (RMSEA) were used as approximate fit indexes (AFIs). Greater CFI and TLI values and smaller RMSEA values indicate better fit. For the CFI and TLI a cut-off point of .95 is used to indicate good model fit, while values greater than .90 are often used to indicate acceptable model fit. For the RMSEA values smaller than .06 indicate good fit [54,55]. Given the large sample size in the current study the χ^2^ was not interpreted as it was shown to be highly sensitive for sample size, but was reported for sake of completeness [55–57].

The first step consisted of testing the overall model fit. Therefore, the hypothesized CFA model was fitted to the combined data of the two settings. This step was performed to assure that, on average, this model reflected the data properly.

The second step consisted of ME/I testing, regarding gender, within each setting separately. This step also enabled the free estimation of mean scores between gender. To check whether the model fitted adequately for each gender, models were initially fitted separately for males and females [56]. Thereafter, four sequential models were conducted to test increasingly restrictive forms of ME/I [32]: a) Configural (Model 1); all parameters were allowed to be free across groups. b) Metric (Model 2); factor loadings were constrained to be equal across groups. c) Scalar (Model 3); factor loadings and thresholds were constrained to be equal across groups. d) Strict (Model 4); factor loadings, thresholds and unique factor variances (UFVs) were constrained to be 1 in both groups [58]. For identification purposes the means in Model 2, 3, and 4 of the factors in the first group were constrained to be 0, while they were freely estimated in the second group. In Model 2 and 3 the UFVs were furthermore only freely estimated for the second group, while they were constrained to be 1 in the first group. Additionally Model 2 had the first threshold of each item constrained to be equal across groups, as were all thresholds for the items which were used to set scale for each factor [58,59].

The third step consisted of ME/I testing between the collective and individual setting. This was done analogous to the second step. Model 1 of the third step therefore included the two final models of the second step which were fitted simultaneously to serve as a test configural ME/I. Model 2 constrained, in addition to the constraints already present within settings between genders, factor loadings to be equal across settings. Model 3 additionally constrained the threshold to be equal across settings, while Model 4 also constrained the UFVs to be equal across settings. In addition to these models, which tested ME/I, two additional models were conducted to investigate structural differences between settings. Model 5, therefore, constrained the variances of the same factor to be equal across all four groups (male-individual, male-collective, female-individual, and female-collective). Model 6 constrained means of the same factor to be equal across settings for the same gender (male-individual = male-collective, and female-individual = female-collective).

As all models in each step were nested, it enabled testing whether models differed from each other. If models do not differ from each other the more parsimonious—more strict form of ME/I—should be preferred [60]. As χ^2^, Δχ^2^ was also highly sensitive for sample size and therefore not plausible to interpret for the evaluation of ME/I [56,57]. Instead, ΔAFIs were used to determine ME/I. However, as ΔAFIs have an unknown sampling distribution it is not possible to test whether differences in AFIs are significant [61]. Several articles, using different caveats, therefore investigated the performance of ΔAFIs to detect ME/I. In the current study two guidelines were used to assess the presence of ME/I, as articles proposed substantially different ΔAFIs cut-off points. The conventional criteria by Chen [62] stated that ΔCFI ≤ −.01 ΔRMSEA ≥ .01 indicate substantial differences between models, therefore not supporting ME/I. Meade et al. [56] recommended more conservative cut-off points; ΔCFI ≤ −.002 ΔRMSEA ≥ .007. In accordance with these conservative cut-off points Marsh et al. [63] advocated the use of ΔTLI = 0 as an additional cut-off point to evaluate the presence of ME/I [61]. As ΔAFIs can give mixed results, depend on several aspects, should not be used as clear cut-off points, and guidelines differ greatly, ME/I is almost never a clear cut case, prohibiting irrefutable claims [61,64]. It seems therefore best to facilitate critical judgement regarding ME/I by presenting the used guidelines, notwithstanding interpretation by the authors for the sake of continuation in the article [56].

As there were two modes of assessment (SAQ vs. CSAQ) in the collective setting, it was assessed whether this had any impact on the SDQ scores. A CFA model for the collective setting was therefore conducted including mode of assessment as a covariate for all factors. School level, dichotomized as preparatory vocational secondary education or lower (VMBO or lower) and senior general secondary education or pre-university education (HAVO or VWO), which was known for this setting, correlated strongly with the mode of assessment and was therefore also included as a covariate. The presence of a potential design effect for the collective setting, due to the class-wise assessment, was also assessed. A multilevel model was therefore conducted enabling the estimation of intraclass correlations depicting the level of variance on the class level in relation to the overall variation [65].

To investigate the direction and magnitude of mean differences for the SDQ scores between settings and gender, an ANOVA was performed. Additionally an ANCOVA was also performed (controlling for age and SES). However, only results, from the ANOVA are presented here, since: (1) Adjustment for age and SES *increased* the effect, although marginally, of the setting on the outcome variables. The more conservative (crude) effect was therefore favoured; (2) Crude SDQ scores are often used to compare different study populations or to define cut-off points and are therefore more valuable to report; (3) Correcting for age could be considered ambiguous as the focus was on the grade of the children (second grade), instead of their age. Percentile scores (80% & 90%) were computed for each setting to give an indication of cut-off scores. Corresponding percentages were furthermore given for these cut-off scores. As a last step the cut-off points from the collective setting were used to categorize the individual sample, in order to investigate the effect of transferring cut-off scores between settings. Analyses were performed using R (version 3.0.2), in combination with Mplus (version 6.11) for the CFA.

## Results

### Missing data

In accordance with Niclasen et al. [10] cases with a total of more than one missing value on all SDQ items were excluded (individual setting: *n* = 170, 2.58%; collective setting: *n* = 50, 1.08%). This handling of missing data was furthermore supported by the notion of Kline [55] that less than 5% of missing data on a single variable is of little concern (range = 0.63–2.31%). The majority of the excluded cases had missings on all items (individual setting = 67.06%, collective setting = 54.00%). It was therefore not appropriate to conduct missing data analysis, as for the majority of the excluded cases background characteristic were also missing. Cases were furthermore deleted when information on gender was not known (individual setting: *n* = 2, 0.03%; collective setting: *n* = 3, 0.07%).

### Background characteristics

Table 1 shows the descriptive statistics for the two settings. There were some small differences regarding the ethnic background, home situation, and domestic area between the settings (Table 1). The mean age was higher in the individual setting compared to the collective setting (Cohen’s d = .76). The average SES was furthermore somewhat higher in the individual setting compared to the collective setting (Cohen’s d = .14).

**Table 1:** Background characteristics for the samples of the individual and collective setting.

	Individual setting	Collective setting	Difference test
N	6,422	4,560	
**Gender** (%)			
Male	48.72	49.78	χ^2^(1) = 1.15, *p* = .28
Female	51.28	50.22	
**Age**			
Mean (SD)	14.26 (0.62)	13.81 (0.57)	t(10,978) = 39.36, *p* < .001
**Ethnic background** (%)			
Dutch	84.56	81.51	χ^2^(2) = 18.69, *p* < .001
Western	8.54	10.64	
Non-Western	6.91	7.86	
**Social economic status**			
Mean (SD)	−0.33 (1.13)	−0.49 (1.13)	t(10,953) = 7.40, *p* < .001
**Home situation** (%)			
2 biological parents	77.95	75.48	χ^2^(2) = 66.86, *p* < .001
1 biological parent	20.74	20.84	
Other/unknown	1.31	3.68	
**Domestic area** (%)			
Rural	51.7	47.66	χ^2^(1) = 13.74, *p* < .001
Urban	48.3	52.34	

### Reliability

Cronbach’s α and glb were calculated for each subscale and the total difficulties scale. Both the total difficulties scale (Cronbach’s α-glb = .78–.85) and hyperactivity/inattention subscale (.76–.81) showed high estimates. The pro-social behaviour (.65–.67) and emotional symptoms subscales (.69–.71) showed moderate estimates, while reliability coefficients for the conduct problems (.55–.56) and peer problems (.51–.55) subscale were low. Reliability coefficients were also calculated for each setting, separately for males and females (Table 2). On average, reliability coefficients were equal across setting and gender and along the lines of the overall estimates.

**Table 2:** Internal consistency of the SDQ scales and standardized factor loadings of the CFA for the 5-factor model with positive construal factor for each setting and gender separately.

	Individual setting		Collective setting	
Scale/Factor	Male	Female	Male	Female
**Total difficulties** (Cronbach’s α–glb)	.75–.84	.78–.86	.77–.85	.76–.85
**Emotional symptoms** (Cronbach’s α–glb)	.59–.61	.68–.72	.62–.64	.68–.72
Somatic	.44	.54	.52	.54
Worries	.62	.67	.59	.60
Unhappy	.78	.82	.80	.83
Clingy	.66	.61	.56	.59
Fears	.67	.66	.72	.64
**Conduct problems** (Cronbach’s α–glb)	.50–.53	.49–.50	.57–.59	.53–.54
Tempers	.65	.67	.69	.66
Obedient	−.08†	−.03†	.21	-.01†
Fights	.55	.56	.66	.65
Lies	.72	.75	.70	.73
Steals	.54	.49	.66	.53
**Hyperactivity** (Cronbach’s α–glb)	.77–.81	.77–.82	.71–.78	.75–.81
Restless	.78	.72	.75	.76
Fidgety	.73	.72	.76	.72
Distractible	.83	.87	.76	.82
Think before acting	.31	.27	.51	.36
Persistent	.47	.61	.54	.52
**Peer problems** (Cronbach’s α–glb)	.51–.55	.52–.57	.51–.56	.46–.50
Solitary	.59	.56	.51	.50
Good friend	.40	.36	.47	.20
Popular	.50	.56	.65	.47
Bullied	.77	.82	.83	.76
Adults	.55	.56	.59	.56
**Pro-social behaviour** (Cronbach’s α–glb)	.59–.61	.56–.58	.65–.68	.64–.67
Considerate	.81	.82	.70	.79
Shares	.38	.43	.43	.42
Caring	.57	.57	.65	.63
Kind to kids	.61	.63	.67	.69
Often volunteers to help	.57	.62	.58	.61
**Positive construal**				
Obedient	.69	.66	.52	.49
Think before acting	.48	.46	.49	.40
Persistent	.31	.25	.46	.27
Good friend	.08†	.03†	.58	.22
Popular	.35	.28	.68	.35

Note: First item of each factor was used as indicator for the CFA; glb = greatest lower bound.

† = not significant at α of .05.

### CFA

CFA were conducted to test for ME/I. The first step consisted of testing the overall model fit for the two settings combined. All factor loadings from the original 5-factor were significant (*p* < .05) and positive, but the model had a mediocre fit, χ^2^ (265) = 8,968.77, *p* < .001, CFI = .8827, TLI = .8672, RMSEA = .0547. In accordance with Roy et al. [9] an extra factor was therefore added comprising the five positively worded items. This resulted in a better fitting model, Δχ^2^ (10) = 1,815.93, *p* < .001, ΔCFI = −.0513, ΔTLI = −.0551, ΔRMSEA = .0129, with an overall satisfactory fit, χ^2^ (255) = 5,153.24, *p* < .001, CFI = .9340, TLI = .9223, RMSEA = .0418. All factor loadings were significant (*p* < .05) and in the expected, positive, direction. The positive construal factor was allowed to correlate with all other factors. For the rest of the analysis this adjusted model was used.

In the second step the model was fitted separately for each setting, to test for ME/I regarding gender within settings. For the individual setting the model had a satisfactory fit for females and males. Factor loadings of these models are given in Table 2. The factor loadings for the *obedient* item on the conduct factor and the *good friend* item on the positive construal factor were non-significant, for both genders, while all other loadings were significant (*p* < .05) and positive. Model 1 estimated these two models conjointly, which showed that configural ME/I hold as model fit was satisfactory to good (Table 3). Metric ME/I was also supported, as constraining the factor loadings did not surpass any ΔAFIs criteria. Model 3, constraining the thresholds of the same items to be equal, only marginally surpassed the conservative criteria regarding the difference in CFI and TLI. The ΔRMSEA was however within these conservative limits. As all conventional criteria were furthermore met, there was no evidence for a strong violation of scalar ME/I. Strict ME/I, constraining the UFVs to be equal across groups, was also supported for the individual setting.

**Table 3:** Model fit and nested model comparisons for multiple-group CFA analyses.

	Model fit indices				Nested model comparisons				
Model (Constraints)	χ^2^ (df)	CFI	TLI	RMSEA	Comp	Δχ^2^ (df)	ΔCFI	ΔTLI	ΔRMSEA
**Individual setting**									
Male (*n* = 3,129)	1,349.51 (255)*	.9385	.9276	.0370					
Female (*n* = 3,293)	1,698.97 (255)*	.9343	.9227	.0415					
1. All parameters free	3,046.03 (510)*	.9363	.9250	.0394					
2. M.1 + Factor loadings	3,009.20 (533)*	.9378	.9300	.0380	vs. 2	91.40 (23)*	.0015	.0049	−.0013
3. M.2 + Thresholds	3,132.62 (553)*	.9352	.9297	.0381	vs. 3	167.74 (20)*	−.0026	−.0003	.0001
4. M.3 + UFVs	3,187.98 (578)*	.9344	.9319	.0375	vs. 4	120.41 (25)*	−.0008	.0022	−.0006
**Collective setting**									
Male (*n* = 2,270)	1,316.38 (255)*	.9209	.9070	.0428					
Female (*n* = 2,290)	1,465.97 (255)*	.9157	.9008	.0455					
1. All parameters free	2,783.67 (510)*	.9180	.9036	.0442					
2. M.1 + Factor loadings	2,797.13 (533)*	.9184	.9081	.0432	vs. 2	92.61 (23)*	.0003	.0045	−.0011
3. M.2 + Thresholds	2,876.39 (553)*	.9163	.9091	.0429	vs. 3	123.83 (20)*	−.0021	.001	−.0002
4. M.3 + UFVs	2,968.76 (578)*	.9138	.9105	.0426	vs. 4	149.34 (25)*	−.0024	.0014	−.0003
**Settings combined (Individual + Collective)**									
1. All parameters free	6,157.52 (1,156)*	.9256	.9228	.0397					
2. M.1 + Factor loadings	6,341.22 (1,179)*	.9232	.9218	.0399	vs. 1	207.86 (23)*	−.0024	−.0009	.0002
3. M.2 + Thresholds	6,489.06 (1,199)*	.9213	.9212	.0401	vs. 2	186.85 (20)*	−.0019	−.0006	.0002
4. M.3 + UFVs	6,790.19 (1,224)*	.9172	.9188	.0407	vs. 3	332.20 (25)*	−.0041	−.0024	.0006
5. M.4 + Factor variances	6,715.55 (1,242)*	.9186	.9213	.0401	vs. 4	84.36 (18)*	.0014	.0025	−.0006
6. M.5 + Factor means	10,348.80 (1,254)*	.8647	.8705	.0514	vs. 5	1,581.83 (12)*	−.0539	−.0508	.0113

Note: * = significant at α of .05; df = degrees of freedom; CFI = Comparative Fit Index; TLI = Tucker-Lewis Index; RMSEA = Root Mean Square Error of Approximation; Comp = Compared to model; UFVs = unique factor variances.

For the collective setting separate model testing for males and females resulted in satisfactory fitting models. Factor loadings of these models are given in Table 2. Only the factor loading for the *obedient* item on the conduct factor for females was non-significant, while all other loadings were significant (*p* < .05) and positive. Joint estimation of these models supported the presence of configural ME/I, as the model fit of Model 1 was satisfactory. Constraining the factor loadings in Model 2 showed the presence of metric ME/I, as all ΔAFIs were within the conservative criteria. Scalar ME/I also seemed to hold for the collective setting, as constraining the threshold to be equal across gender resulted in an acceptable change of model fit. Only the ΔCFI marginally surpassed the conservative criteria. This was also found when the UFVs were constrained to be 1 in both groups, supporting strict ME/I (Table 3). As the CFA models of both settings showed good fit and supported ME/I, regarding gender, it was justified to proceed to the third step in which ME/I between settings was tested.

In the third step the collective setting and individuals setting models were jointly estimated to assess ME/I. Model 1 showed satisfactory fit supporting configural ME/I. Metric ME/I was supported according to the conventional criteria and the conservative ΔRMSEA criteria, this indicated that factor loadings were similar across settings. The test for scalar ME/I resulted in the same conclusion, with also the ΔCFI falling within the conservative criteria. This indicated that thresholds of the SDQ items were equal across the collective and individual setting. Constraining the UFVs to be equal across settings resulted in a change of model fit surpassing the conservative criteria regarding the ΔCFI and ΔTLI. Changes were, however, within the conventional criteria and the conservative ΔRMSEA criteria indicating that there was no strong violation of strict ME/I (Table 3). As metric and scalar ME/I were furthermore supported, testing structural differences between settings was legitimate.

To test for structural differences, additional constraints were added to Model 4 of the combined estimation (Table 3). First, variances of the same factors were set to be equal across both genders for both settings. This resulted in a model change falling within the conservative criteria, supporting that there were no differences in the variances of any factor across settings or gender. In the last model, factor means were additionally constrained to be equal for the same gender across settings. This implied that the average scores of the same gender across settings were approximately equal for all SDQ factors. The decrease in model fit implied, however, the presence of structural differences between settings regarding mean scores.

To test for a potential mode effect, SAQ vs. CSAQ, a CFA model for the collective setting, female and male combined, was conducted with mode of assessment and school level as covariates of all factors. This resulted in a good fitting model, χ^2^ (293) = 2,620.90, *p* < .001, CFI = .9180, TLI = .9020, RMSEA = .0420, with no statistically significant association between mode of assessment and any SDQ-factor (*p* > .05). The presence of a design effect, through classes, in the collective setting was assessed with a multilevel model. This multilevel model showed a good model fit χ^2^ (510) = 4,248.96, *p* < .001, CFI = .9260, TLI = .9130, RMSEA = .0410. Intraclass correlations for all factors (range = .02–.08) and items (range = .00–.07) were low, indicating that no more than 7.81% of the variance in any factor was due to the class-wise assessment.

### Mean Scores

To investigate the structural mean differences of the subscales, and the total difficulties score, an ANOVA was performed (Table 4). Setting was significant (*p* < .05) for all scale scores, with a small association for hyperactivity and a more moderate effect for pro-social behaviour, conduct problems and the total difficulties score. Scores from the collective setting were higher than the scores from the individual setting, except for the pro-social behaviour subscale. However as this subscale was an index of positive behaviour, the direction of the effect of setting was the same for each subscale and the total difficulties score.

**Table 4:** Comparisons for SDQ total difficulties and subscales mean scores for each setting and gender.

	Setting; mean (SD)		η^2^ (effect size)		
Scale	Individual setting	Collective setting	Setting	Gender	Interaction
**Total difficulties score**			.04*	.01*	.002*
Males	7.31^a^ (4.49)	8.96^b^ (5.12)			
Females	7.81^c^ (4.83)	10.27^d^ (5.20)			
**Emotional symptoms**			.02*	.09*	.004*
Males	1.42^e^ (1.54)	1.66^f^ (1.77)			
Females	2.40^g^ (2.05)	3.12^h^ (2.29)			
**Conduct problems**			.05*	.005*	< .001
Males	1.27 (1.26)	1.89 (1.65)			
Females	1.09 (1.14)	1.70 (1.44)			
**Hyperactivity**			.01*	< .001	.001*
Males	3.58^i^ (2.46)	3.93^j^ (2.43)			
Females	3.35^k^ (2.38)	4.08^j^ (2.45)			
**Peer problems**			.02*	.001*	< .001
Males	1.04 (1.33)	1.47 (1.60)			
Females	0.97 (1.30)	1.37 (1.48)			
**Pro-social behaviour**			.05*	.06*	.001*
Males	7.92^l^ (1.62)	7.02^m^ (2.00)			
Females	8.67^n^ (1.34)	7.95^l^ (1.73)			

Note: * = significant at α of .05; If the interaction effect was significant for a scale a post-hoc test was performed between the four groups, in which groups with different superscripts differed significantly from each other, while equal superscripts indicate no significant difference between groups. If the interaction effect was not significant for a scale a post-hoc test was not performed.

Gender was significantly (*p* < .05) associated with all scale scores except hyperactivity. Interaction effects were small, but nonetheless significant (*p* < .05) for emotional symptoms, hyperactivity, pro-social behaviour and total difficulties score. Inspection of Table 4 shows that if an interaction effect was present, females showed larger differences between settings than males.

The ANCOVA included age and SES, which strongly relates with domestic area and home situation, as covariates. The overall effect of age (in years) on the total difficulties score was b = 0.68 (*p* < .01). The individual setting showed a somewhat smaller effect, b = 0.48 (*p* < .01), while the effect in the collective setting was higher, b = 1.01 (*p* < .01). The overall effect of SES was not significant at b = -0.04 (*p* = .29), which remained when analysed separately for both groups (individual setting, b = 0.08 [*p* = .08], collective setting, b = -0.09 [*p* = .84]). Subsequently, an ANCOVA was performed, with age and SES as covariates, using the overall effects as correction. The effect size of the setting only marginally increased while there was no change in the effect size of gender and the interaction term (gender × setting). The effects of the covariates on the total difficulties score were generalizable to all subscales, except the prosocial subscale which showed the inverse pattern. As indicated in the methods section the results of the ANOVA were favoured above those of the ANCOVA.

### Cut-off points

To further illustrate the impact of the structural differences between the settings, cut-off points were calculated for the current samples, separately for each setting. As shown in Table 5 all cut-off points were lower in the individual setting as compared to the collective setting. Differences between females and males were in accordance with the differences in mean scores as found in the ANOVA. There were for instance more females with a borderline or abnormal score regarding hyperactivity in the individual setting, while in the collective setting these cases were more prevalent among males.

**Table 5:** SDQ cut-off scores and percentage of cases for the individual and collective setting based on the 80^th^ and 90^th^ percentile of these settings and the percentage of cases in the individual setting based on the cut-off scores in the collective setting.

	Normal: 0–80 percentile				Borderline: 80–90 percentile				Abnormal: >90 percentile			
Scale	Cut-off	T%	M%	F%	Cut-off	T%	M%	F%	Cut-off	T%	M%	F%
**Collective setting**												
Total difficulties score	0–14	83	86	79	15–17	9	7	11	18–40	9	7	10
Emotional symptoms	0–4	83	91	74	5–6	12	7	16	7–10	6	2	10
Conduct problems	0–3	87	86	89	4	6	6	6	5–10	7	8	5
Hyperactivity	0–6	84	85	83	7	6	6	7	8–10	10	9	10
Peer problems	0–3	89	88	91	4	6	7	5	5–10	5	6	4
Pro-social behaviour	10–6	85	79	90	5	8	11	6	4–0	7	10	4
**Individual setting**												
Total difficulties score	0–11	82	83	80	12–14	10	10	10	15–40	8	7	10
Emotional symptoms	0–3	82	90	74	4–5	13	8	18	6–10	5	2	8
Conduct problems	0–2	88	86	90	3	7	9	6	4–10	5	6	4
Hyperactivity	0–5	80	79	82	6–7	13	14	12	8–10	7	8	6
Peer problems	0–2	87	87	88	3	7	7	6	4–10	6	6	5
Pro-social behaviour	10–7	87	81	93	6	7	10	4	5–0	6	8	3
**Individual setting using cut-off scores from Collective setting**												
Total difficulties score	0–14	92	93	90	15–17	5	4	6	18–40	3	2	4
Emotional symptoms	0–4	90	95	84	5–6	7	4	11	7–10	3	1	5
Conduct problems	0–3	95	94	96	4	3	3	3	5–10	2	3	1
Hyperactivity	0–6	88	86	89	7	6	6	5	8–10	7	8	6
Peer problems	0–3	94	94	95	4	3	3	3	5–10	2	3	2
Pro-social behaviour	10–6	94	92	97	5	4	6	2	4–0	2	3	1

Note: T = Total; M = Male; F = Female

As in daily practice cut-off scores from collective settings are often applied in individual settings, this process was simulated using the current data. The sample from the individual setting was therefore differentiated in abnormal, borderline, and normal cases using the cut-off scores from the collective setting (Table 5). This resulted in much lower prevalence rates of abnormal case on all scales, except hyperactivity. On the total difficulties score for instance only 8% of the children were classified as a borderline or abnormal case, instead of the original 18%. If only abnormal cases were selected this percentage even drops to 3% instead of the original 8%.

## Discussion

Using an observational approach, this study investigated whether assessment of the self-report version of the SDQ is sensitive to its administration setting. Using two large samples results show that, although the SDQ has the same connotation across both settings, there are significant structural differences between the collective and individual setting.

**Fig 1:**
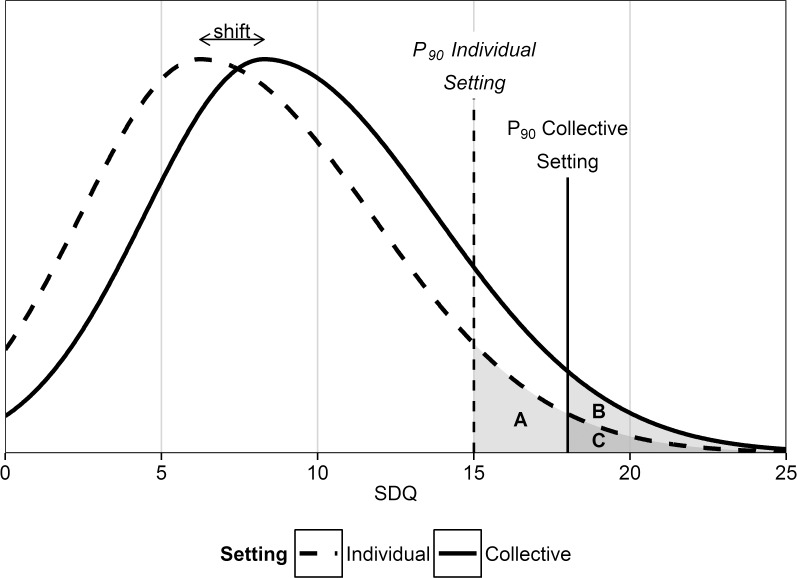
Theoretical density plots for the distributions of the SDQ total difficulties scores for the collective and individual setting, illustrating the shift between the two settings. 90^th^ percentile cut-off points are given for both settings. Segments illustrate children falling within the abnormal range (∼10%) of the individual (A+C) and collective (B+C) setting using the respective cut-off points. Children in the individual setting falling within segment C have extreme scores, defined by the cut-off point of the collective setting.

Structural differences complicate the cross-use of cut-off points between settings. In the individual setting these cut-off points facilitate the screening purpose of the SDQ to differentiate between children with and without an elevated risk for developing psychopathology. Cut-off points in individual settings are often retrieved from validation studies, which are typically conducted in collective settings. There are, however, structural differences between the mean scores of the subscales and total difficulties score of the SDQ while ME/I and equal factor variances hold. These three findings combined imply that the sample distribution of the SDQ has the same form and represents the same construct for both settings, but shifts, as a whole, between the two settings (Fig. 1). The cross-use of cut-off scores between settings is therefore problematic. If, for example, the cut-off score from the collective setting, based on its 90^th^ percentile (segment B + C; Fig. 1), is applied in the individual setting only 3% of the scores are abnormal (segment C). Only the most extreme scores are, therefore, seen as abnormal instead of the expected ∼10% (segment A + C). Predictive validity studies are based on these ∼10% highest scores (B + C). Children falling within the most extreme scores (segment C) are, however, more at risk for psychopathology compared to children who do not score as high but do score above the individual 90^th^ percentile (segment A), as previous research indicates that an increase on the SDQ across the full range is related with increased odds for psychopathology [66]. Using the cut-off score from the collective setting in the individual setting would therefore increase the ratio of children who are correctly characterized as a case, true positives, compared to those incorrectly characterized as a case, false positives, (i.e. resulting in an increased positive predictive value) while the ratio of children who are correctly characterized as a non-case, true negatives, compared to those incorrectly characterized as a non-case, false negatives, would decrease (i.e. resulting in a decreased negative predictive value). This would bias the interpretation of caseness, as the chance that a child, scoring above the cut-off point, develops psychopathology is higher than expected. For an informed and valid usage of the SDQ in the individual setting the “actual” 90^th^ percentile cut-off has to be determined. Or, the accuracy in this individual setting has to be reassessed using the collective setting cut-off scores.

For the collective setting these results show that it is important that attention is paid to the setting of the original assessment. As Mieloo et al. [67] point out in their discussion regarding their use of SDQ scores retrieved from an individual setting: “Thus, generalizing our findings to an anonymous [collective] research setting probably requires caution (p. 7).” Based on the results of the present article this caution seems warranted, as the individual setting may yield an underestimation of SDQ scores compared to the collective setting. Besides the possible underestimation of SDQ scores, and associated caseness, interaction effects between gender and setting adds another potential hazard. No significant difference, for instance, is present on the hyperactivity subscale in the collective setting whilst there is in the individual setting, with males scoring higher than females. The magnitude, or even direction, of differences between groups, such as gender, can therefore depend on the assessment setting. Although structural mean differences prevent the cross-use of absolute scores (e.g. cut-off and mean scores), relative scores (e.g. trends and *z*-scores) are comparable between settings as the SDQ has the same meaning in both settings. While the assessment setting showed structural differences, the present study showed no differences between scores derived from administration through CSAQ or SAQ. Overall, these findings illustrate the importance of taking sources of variation regarding the mode of assessment (e.g. interview versus self-administration) into account when interpreting or comparing the results of studies [68].

The direction of the structural differences between the settings is as hypothesized based on the literature regarding SDB. For all scores, except the pro-social subscale, mean scores of the individual setting are lower compared to the collective, setting. This enhanced SDB is likely to be a result of the confidential nature of the individual setting, compared to the anonymous nature of the collective setting, in combination with the potential desirability to remain ”off the radar” as high scores can have personal consequences [27]. The impact of SDB was however not limited to the understating of negative behaviour, but also resulted in the exaggeration of positive behaviour. This is shown by the higher scores on the pro-social subscale in the individual setting compared to the collective setting. It is furthermore noteworthy that not only an effect is present for the more sensitive questions, regarding conduct problems, but also for the relative insensitive questions regarding hyperactivity behaviour. This shows that it remains difficult to determine which questions are sensitive and which are not, especially for youths [27]. Previous research for example found that substance use seems relatively unaffected by the level of anonymity, whilst such an effect has been more readily established for the disclosement of sexual and antisocial behaviour [25,29,69]. Also in line with previous research is the finding that females show more profound differences on sensitive health indicators between varying levels of anonymity, compared to males [29,30].

CFA show that ME/I seems to hold between the settings and within each setting regarding gender. Mean comparisons, between gender and settings, regarding the SDQ scores and interpretation of the direction of the SDB are therefore justified as they are not an artefact of measurement bias. It furthermore validates the extrapolation of conclusions between settings, such as the established relation between the SDQ and psychopathology—the basis for its usage in the individual setting [15]. The establishment of metric ME/I also supports the absence of a difference regarding extreme response styles (ERS), between the settings [70]. Despite the elevated SDB, there is therefore no excessive reliance on the extreme categories in the individual setting compared to the collective setting.

For both the individual and collective setting this article shows that the SDQ is a reliable instrument to use. As previous research also shows, the total difficulties score should be preferred to the subscales [8]. This is due to the reliability of some subscales. Especially the conduct problems subscale scores low, the reliability of the peer problems, pro-social, and emotional symptoms subscales is however also below preferred levels. Favouring the total difficulties score above the subscales is in line with the primary purpose of the SDQ as an early screening instrument. That is, although the SDQ can differentiate between different psychopathologies (e.g. attention deficit hyperactivity disorder vs. obsessive–compulsive disorder), it is by no means a psychiatric diagnostic instrument [14,15].

Some strengths and limitations of the present study should be noted. The sample size from both samples was large and the amount of missingness was very small. In contrast to most studies investigating SDB, the current study had furthermore an observational design instead of an experimental. It is, however, not possible to capture the true nature of the individual setting in an experimental design. Samples from both settings represent, however, the same socio-geographic population. Some, mostly small, differences on the background characteristics, nevertheless, occurred. Controlling for SES and age did, however, only marginally influence the results and, if anything, resulted in a larger effect size for setting. Comparison seems therefore valid, despite the difference in selection procedures (opt-in vs. opt-out), response rates, and background characteristics between the two settings. It could, furthermore, be argued that differences between the two settings are a characteristic of the settings as they illustrate the true nature of such settings. Although the sample size was large and the sample heterogeneous, the presented cut-off points, both for the collective and individual setting, should not be used as cut-off points for any of these two settings since the study was limited to a specific socio-geographic population. These cut-off points however do illustrate the mechanisms that will be present when cut-off points from actual, collective setting, validation studies will be used in the individual setting.

Regarding the CFA models it has to be noted, that since the original 5-factor structure proposed by Goodman [15] shows a mediocre fit an adaptation of this structure was used for all following models which shows good fit throughout. This adaptation includes a positive construal factor for all positively worded items [71]. The increased fit supports previous research advocating inclusion of this factor [9,72]. Alternative factor models can however not be ruled out as they are not conducted as this was beyond the focus of this article. There is furthermore no clear consensus regarding the CFA procedure for establishing ME/I, especially regarding categorical variables and the use of ΔAFI for this purpose [32,61,73]. By providing and interpreting multiple ΔAFI along two common guidelines from the literature, the process is presented in a transparent and insightful manner [56,62].

To conclude, this study shows that the SDQ has the same meaning when measuring psychological problems in children in a collective and in an individual setting. Due to the SDB, seemingly inflicted by the confidential nature and possible personal consequences of high scores in the individual setting, there are however structural differences between the settings. These structural differences result in invalid mean comparisons and cross-use of cut-off points between settings and should be taken into account in future studies and should also be considered when interpreting finding from earlier studies. One should, therefore, be aware of the potential pitfalls that are instigated by the structural differences between the settings. For the collective setting this implies that (cross-country) comparisons of absolute scores between samples could be seriously hampered if the setting of assessment differs, while for the individual setting these structural differences result in an increased positive predictive value and decreased negative predictive value when cut-off points from the collective setting are used (Fig. 1). Although the use of cut-off points on itself will always be debated, it is crucial that the SDQ works in the way it is “advertised” achieving a certain accuracy [66]. It is therefore of the utmost importance that if cut-off scores are used they are retrieved from the same setting as they are applied in.
